# Transcriptome Analysis Reveals Unfolded Protein Response Was Induced During the Early Stage of *Burkholderia pseudomallei* Infection in A549 Cells

**DOI:** 10.3389/fgene.2020.585203

**Published:** 2020-12-08

**Authors:** Chenglong Rao, Chan Mao, Yupei Xia, Meijuan Zhang, Zhiqiang Hu, Siqi Yuan, Wenbo Yang, Jingmin Yan, Ling Deng, Xiaolian Cai, Xuhu Mao, Qian Li, Yaling Liao

**Affiliations:** ^1^Department of Clinical Microbiology and Immunology, College of Pharmacy and Medical Laboratory, Army Medical University (Third Military Medical University), Chongqing, China; ^2^Department of Pharmacy, Second Affiliated Hospital, Army Medical University (Third Military Medical University), Chongqing, China; ^3^Department of Cardiology, First Affiliated Hospital, Army Medical University (Third Military Medical University), Chongqing, China

**Keywords:** *Burkholderia pseudomallei*, transcriptomics, unfolded protein response (UPR), protein processing in endoplasmic reticulum, differential expression genes (DEGs)

## Abstract

*Burkholderia pseudomallei* is a zoonotic pathogen that usually affects patients' lungs and causes serious melioidosis. The interaction of *B. pseudomallei* with its hosts is complex, and cellular response to *B. pseudomallei* infection in humans still remains to be elucidated. In this study, transcriptomic profiling of *B. pseudomallei*-infected human lung epithelial A549 cells was performed to characterize the cellular response dynamics during the early infection (EI) stage. Gene Ontology (GO) and Kyoto Encyclopedia of Genes and Genomes (KEGG) pathway analyses were performed by using the online databases DAVID 6.8 and KOBAS 3.0. Real-time quantitative PCR and western blot were used for validation experiments. Compared with the negative control group (NC), a set of 36 common genes varied over time with a cut-off level of 1.5-fold change, and a *P*-value < 0.05 was identified. Bioinformatics analysis indicated that the PERK-mediated unfolded protein response (UPR) was enriched as the most noteworthy biological process category, which was enriched as a branch of UPR in the signaling pathway of protein processing in the endoplasmic reticulum. Other categories, such as inflammatory responses, cell migration, and apoptosis, were also focused. The molecular chaperone Bip (GRP78), PERK, and PERK sensor-dependent phosphorylation of eIF2α (p-eIF2α) and ATF4 were verified to be increasing over time during the EI stage, suggesting that *B. pseudomallei* infection activated the PERK-mediated UPR in A549 cells. Collectively, these results provide important initial insights into the intimate interaction between *B. pseudomallei* and lung epithelial cells, which can be further explored toward the elucidation of the cellular mechanisms of *B. pseudomallei* infections in humans.

## Introduction

Melioidosis is a potentially fatal infectious disease in tropical and subtropical countries worldwide caused by the Gram-negative bacteria *Burkholderia pseudomallei* (Wiersinga et al., 2012). Humans and animals usually acquire this disease through broken skin, inhalation, or ingestion of the Tier-1 (top tier) select agent *B. pseudomallei* (Wiersinga et al., 2006). It is estimated that there are ~165,000 human melioidosis cases and ~89,000 deaths (54%) worldwide per year (Limmathurotsakul et al., 2016). Due to the globalization of the world's tourism and trade, the epidemic areas are expanding (Wiersinga et al., 2012, 2018; Limmathurotsakul et al., 2016). In addition, there is no licensed vaccine for the prevention of disease (Titball et al., 2017). It is particularly important and prominent to make a profound study on the pathogenic mechanism of *B. pseudomallei*.

*B. pseudomallei* is a facultative intracellular bacterium, and it can adhere to and invade a number of mammalian cell types (Jones et al., 1996; Williams et al., 2014) and persist *in vivo* for many years (Mays and Ricketts, 1975). During the early infection (EI) stage, *B. pseudomallei* can escape from the endocytic vacuole into the cytosol, next modulate different cellular responses, and evade intracellular killing in infected cells (Willcocks et al., 2016). Previous studies have shown that *B. pseudomallei* modulated host iron homeostasis to facilitate iron availability and intracellular survival (Schmidt et al., 2018). It could also evade LC3-associated phagocytosis (Gong et al., 2011) and induce macrophages pyroptosis (Bast et al., 2014). Our previous research demonstrated that *B. pseudomallei* could interfere with the progress of Rab32 GTPase-mediated *B. pseudomallei*-containing phagosomes maturation and escape into the cytosol (Hu et al., 2019). *B. pseudomallei* could evade autophagy by regulating ATG10 in A549 cells (Li et al., 2015). Moreover, *B. pseudomallei* acquired the evolutionary ability to subvert autophagy by hijacking host lipid metabolism for intracellular survival (Tang et al., 2020). However, the interaction of *B. pseudomallei* with its hosts is more complex, and cellular response to *B. pseudomallei* infection in humans still remains incomplete.

Transcriptome profiling is an effective strategy for understanding the molecular events in host–pathogen interactions in recent years (Tuanyok et al., 2006; Chin et al., 2010). Previous transcriptome studies about *B. pseudomallei* have shown that it could up- or down-regulate numerous genes to adapt rapidly to the intracellular environment in human macrophage-like U937 cells during the EI stage (Chieng et al., 2012). *B. pseudomallei* could up-regulate two component signal transduction systems and a denitrification enzyme pathway for biofilm production and virulence (Chin et al., 2015; Wong et al., 2015). The small colony variant (SCV) could up-regulate many virulence and survival factors pre- and post-exposed to A549 cells (Al-Maleki et al., 2020). Previous studies are limited by a narrow dynamic expression range, and little is known about the dynamic changes of host response to *B. pseudomallei* infection.

Human lung epithelial cells are particularly susceptible following exposure by inhalation (Zueter et al., 2016). Multinucleated giant cell (MNGC) formation is an important characteristic feature of the intracellular life cycle of *B. pseudomallei* during the late dissemination stage (Whiteley et al., 2017). The persistent infection of *B. pseudomallei* promotes the fusion of an infected mononuclear cell with one or more neighboring cells (Kespichayawattana et al., 2000; McNally and Anderson, 2011). Identifying the differentially expressed genes (DEGs) of host cells and digging their inter-relationships, such as enriched biological processes and pathways, will help to provide insight into the combined influence of *B. pseudomallei* on body function. Therefore, we divided the EI stage according to no more than 50% MNGC formation and dynamically investigated the global transcriptional response of human lung epithelial cell A549 infected with *B. pseudomallei* using microarray analysis during the EI stage to get a better understanding of genes association. Analysis of the DEGs revealed that the response genes of A549 cells to *B. pseudomallei* infection were mainly involved in unfolded protein response (UPR). UPR affects host's proteins synthesis and maturation, and the finding may provide a new direction for future work to reveal the pathogenic mechanism of *B. pseudomallei* in manipulating UPR signaling.

## Materials and Methods

### Cell Lines and Bacterial Strains

Human lung epithelial cell line A549 (ATCC, CCL_185) was grown in Dulbecco's Modified Eagle Medium (DMEM, Gibco, 11965-092) containing 10% fetal bovine serum (Gibco, 10099-141) without the addition of antibiotics at 37°C with an atmosphere containing 5% CO_2_. *B. pseudomallei* C006 (BPC006), as a representative of clinical virulent strain, which was the first sequencing strain in China, was used for bacterial infection in this study (Fang et al., 2012). The genome sequence of BPC006 was very close to the international standard strain K96243, and the mapped reads were ~94% (Supplementary Table 1). BPC006 was cultured in Luria-Bertani (LB) broth at 37°C with shaking at 200 rpm, and the overnight culture was washed twice in phosphate-buffered saline (PBS) and adjusted to an appropriate concentration by measurement of the optical density at 600 nm for infection. Live *B. pseudomallei* was handled under standard laboratory conditions (biosafety containment level 3).

### Immunofluorescence and MNGC Formation

A549 cells were seeded in 12-well plates with 1 × 10^5^ cells per well and grown on glass coverslips (NEXT, China) overnight. Subsequently, the cells were infected with *B. pseudomallei* at a multiplicity of infection (MOI) of 10 and incubated for 6 or 12 h. The cells were then fixed with 4% paraformaldehyde (PFA, Electron Microscopy Sciences) for 10 min and permeabilized with 0.05% Triton X-100 (Sigma, T8787). *B. pseudomallei*-infected fixed cells were incubated with a rabbit polyclonal antibody of *B. pseudo*mallei (1:500) from our previous research (Hu et al., 2019) and subsequently with the Alexa Fluor 568 anti-rabbit secondary antibody (Abcam, UK) (1:2,000). The cells were stained with Actin-Tracker Green (Biotechnology, Shanghai, China). Nuclei were counterstained with DAPI (Life Technologies, 300 nM). Cover glasses were mounted on glass slides using a fluorescence mounting medium (DakoCytomation). Images were acquired on a fluorescence microscope (Zeiss, Germany) using a 40× (60× for *B. pseudomallei*) lens objective.

MNGCs were defined as cells containing at least three nuclei. Counting statistics of MNGCs was conducted according to the method outlined in previous reports (Whiteley et al., 2017). In brief, MNGC formation efficiency (as a percentage) was determined with a 20× objective using the following formula: (N within multinucleated giant cells / total N) × 100, where N is the number of nuclei. A minimum of 2,300 nuclei were counted for each condition. Three independent replicates were performed at each time point in the test.

### Intracellular Survival of Bacteria

Intracellular survival of *B. pseudomallei* in A549 was estimated as previously described with some modifications (Li et al., 2015). In the initial 2 h infection with *B. pseudomallei*, the bacteria and cells were co-incubated for 1 h, and then the cells were overlaid with DMEM containing 250 μg/ml kanamycin for 1 h to kill extracellular bacteria. Subsequently, the cells were washed with PBS and maintained with 20 μg/ml of kanamycin in DMEM for the next 1, 4, 8, 12, 16, and 20 h. After incubation, infected A549 cells were washed three times with PBS and lysed with 1 ml of 0.1% Triton X-100 (Sigma, T8787). Cell lysates were collected and serially diluted 10-fold in PBS, and aliquots of 10 μl were plated onto LB agar to assess viable bacterial counts. We performed the experiment three replicates independently.

### GeneChip™ Microarray Assay

The A549 RNA extracted from 6 to 12 h post-infection (hpi) was isolated by means of the RNeasy Micro Kit (Qiagen) according to the manufacturer's protocol for transcriptome analyses using GeneChip Human Gene 2.0 ST Arrays (Affymetrix). The control wells at 0 hpi without *B. pseudomallei* were used as reference group for normalization. Sample preparation for microarray hybridization was carried out as described in the Affymetrix GeneChip® Whole Transcript (WT) Sense Target Labeling Assay manual (Affymetrix, Inc., Santa Clara, CA, USA). In brief, 300 ng of total RNA was used to generate double-stranded cDNA. First, cRNA was synthesized (WT cDNA Synthesis and Amplification Kit, Affymetrix), purified, and reverse transcribed into single-stranded DNA. Purified ssDNA was then fragmented and labeled with biotin (WT Terminal Labeling Kit, Affymetrix). Finally, 2.3 μg DNA was hybridized to GeneChip Human Gene 2.0 ST Arrays (Affymetrix) for 16 h at 45°C in a rotating chamber. Hybridized arrays were washed and stained in the Affymetrix Washing Station FS450 using Hyb, Wash & Stain Kit (Affymetrix), and the fluorescent signals were measured in the Affymetrix GeneChip® Scanner 3000-7G. Sample processing was performed at the Affymetrix Service Provider and Core Facility, “KFB-Center of Excellence for Fluorescent Bioanalytics” (Regensburg, Germany; http://www.kfb-regensburg.de). For microarray data analysis, the RMA algorithm in the Affymetrix GeneChip Expression Console software 4.0.1 was used to summarize probe signals. Then, they were exported to Microsoft Excel, and average signal values, comparison fold changes, and significance *P*-values were calculated. Probe sets with a fold change above 1.5-fold and a Student's *t*-test *P*-value lower than 0.05 were considered as significantly regulated according to the criteria in a previous report (Manalo et al., 2005).

### Functional Annotation and Enrichment Analysis of DEGs

Gene Ontology (GO) is a functional annotation of the DEGs. The mRNAs exhibiting differential expression were entered into the integrated discovery online server (http://david.abcc.ncifcrf.gov) to be annotated and visualized. The analyses included classifications of cell constituents and molecular function, as well as biological processes, with a confidence level of 95%. Kyoto Encyclopedia of Genes and Genomes (KEGG) pathway analysis using the KEGG Orthology-Based Annotation System (KOBAS; http://kobas.cbi.pku.edu.cn/home.do) was also conducted, and the signaling pathways were verified using the Fisher's test and an false discovery rate (FDR) of ≤0.05. Significant DEGs were used to populate human pathways in KOBAS, with statistical significance determined by Fisher's exact test or hypergeometric test.

### Validation of Microarray Assay

To validate the data generated from GO analysis and pathway analysis, 11 DEGs were selected from different functional categories for real-time quantitative PCR (RT-qPCR) analysis. Supplementary Table 2 shows the respective primer sequences of selected mRNA transcripts. β-Actin and GAPDH were used as reference genes for normalization. The total RNA used for RT-PCR was extracted by TRIzol (Invitrogen Life Technologies) according to the manufacturer's recommendation. Next, complementary DNA (cDNA) was retro-transcribed from 1 μg of total RNA using PrimeScript™ RT reagent kit with gDNA Eraser (Takara, Dalian, China). CFX96 Touch Real-Time PCR detection system (Bio-Rad, USA) was used for PCR detection. The reaction system (total volume: 25 μl) consisted of the following: 12.5 μl SYBR Premix Ex Taq II (Takara, Dalian, China), 1 μl 10 mmol/L upstream primer and 1 μl 10 mmol/L downstream primer, 2 μl of ~50 ng/μl cDNA template, and supplemented with ddH_2_O to a final reaction volume of 25 μl. The PCR program was set as 95°C for 2 min and then 40 cycles of 95°C for 5 s and 60°C for 30 s. To confirm that only one PCR product was amplified, the PCR product was subjected to dissociation curve analysis at the end of each PCR reaction. Each assay was conducted in three replicates. The method of 2^−ΔΔCT^ was used to calculate the relative expression levels of mRNAs in cells after *B. pseudomallei* infection, which were expressed as the relative fold change in the expression level in infected cells divided by that in control cells.

### Western Blot Analysis

The cells from 6 to 12 hpi were collected and lysed in RIPA lysis buffer (50 mM Tris pH 7.4, 150 mM NaCl, 1% Triton X-100, 1% sodium deoxycholate, 0.1% SDS) containing protease and phosphatase inhibitor on ice for 30 min. Cell suspensions were centrifuged at 12,000*g* for 15 min at 4°C, and then cell supernatants were collected and incubated at 100°C for 10 min. Protein concentration was determined by BCA Protein Assay according to the instructions of the supplier (Beyotime, Beijing, China). Equal amounts of protein samples (40 μg) were loaded on 10% SDS-PAGE and electrophoretically transferred onto PVDF membranes (Roche, Switzerland). A commercial protein marker was used for identification of protein size. The membranes were blocked with 5% (w/v) nonfat dry milk for 2 h, followed by an overnight incubation at 4°C with BiP (CST 3177S, 1:1,000), PERK (CST 12703, 1:1,000), Phospho-eIF2α (Ser51, CST 3398, 1:1,000), eIF2α (CST 5324S, 1:1,000), and ATF4 (CST 11815, 1:1,000) antibody, respectively, in which p-eIF2α was an activated form of eIF2α in PERK-mediated UPR. Membranes were subsequently incubated with HRP-linked anti-rabbit IgG secondary antibody (CST 7074S, 1:5,000) at the room temperature of 25°C for 2 h. The bound antibodies were monitored with chemiluminescence using an electrogenerated chemiluminescence detection system (ChemiDoc XRS System, Bio-Rad, USA), and the density of each protein band was quantified using Image Lab software 6.0 (Bio-Rad Laboratories). GAPDH (CST 8884, 1:1,000) and β-actin (CST 3700, 1:5,000) were used as loading controls, and three independent replicates were performed.

### Statistical Analysis

Statistical analysis of gene expression was performed by means of Student's *t*-test. SPSS software for Windows (IBM, V.20.0) was used. Statistical significance was stated in case of *P*-values being lower than 0.05.

## Results

### MNGC Formation

**Figure 1:**
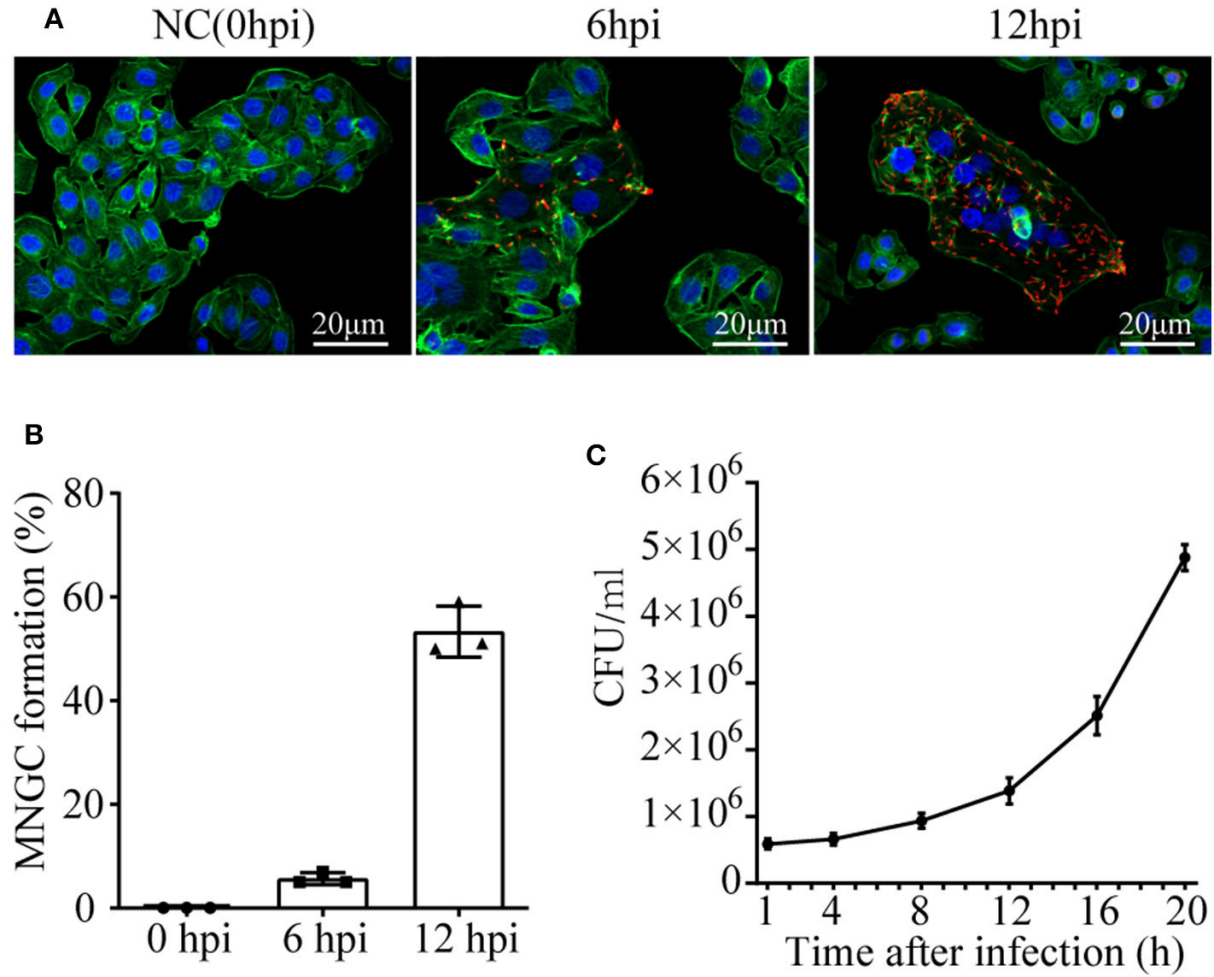
MNGC formation and *B. pseudomallei* intracellular growth in A549 cells. **(A)** Confocal microscope shows the formation of MNGCs induced by *B. pseudomallei* at different infected times. Red is the *B. pseudomallei* indicated by Alexa Fluor 568, blue is the nucleus indicated by DAPI, and green is the cytoskeleton protein actin indicated by Actin-Tracker Green. **(B)** The percentage of MNGC formation was shown at 0, 6, and 12 hpi. **(C)** Intracellular growth of *B. pseudomallei* in A549 cell lines was assayed at different time points (1–20 h) following the initial 2 h infection with a MOI of 10. Three replicates were used for each time point. Bars represent the standard deviation of three replicates.

To observe the cell morphology, cytoskeleton protein actin was stained with Actin-Tracker Green, and confocal microscope was employed. A time series assessment of A549 phenotype challenged with *B. pseudomallei* was shown in Figure 1A. Three independent replicates were performed for each time point in the test. There were no observable microscopic reaction lesions at 0 hpi. By 6 hpi, MNGCs had appeared with a percentage of 5.67 ± 1.15, and most of the infected cells did not fuse at this period, maintaining normal cell morphology with clear cell boundary and intact outline. However, at 12 hpi, the MNGCs had developed much larger, and the percentage increased up to 53.33 ± 4.935 (Figure 1B). Actually, the infection of *B. pseudomallei* was a continuous process. The early and late stages of *B. pseudomallei* infection were not strictly defined. We divide the early stage of infection based on the formation of MNGCs no more than 50% for the convenience of research. Therefore, the results indicated that the early stage of infection could be determined before 12 hpi.

### *B. pseudomallei* Intracellular Growth

Taking the infected time as the abscissa axis and the number of viable bacteria as the ordinate axis, growth kinetics curve was drawn in Figure 1C. The ability of *B. pseudomallei* to survive and replicate intracellularly demonstrated a slow increase from 1 to 12 hpi, with a growth rate of about 9 × 10^4^ cfu/h. However, during 12–20 hpi, the growth of intracellular *B. pseudomallei* was rapid, with a high rate of about 4 × 10^5^ cfu/h. Combined with intracellular *B. pseudomallei* in Figure 1A, the intracellular number at 6 hpi was much lower than that at 12 hpi. It should be mentioned that the morphology of the majority of infected cells at 6 hpi still remained normal. On the basis of all the above results, it could be deduced that the time of 6 hpi was similar to the lag phase of intracellular *B. pseudomallei*, that the bacteria were in the internalization stage, and that the time of 12 hpi was similar to the intracellular multiplication stage. We further choose the two time points for subsequent dynamic transcriptome research to appropriately reveal transcriptomic profiles of A549 with *B. pseudomallei* infection during the EI stage.

### Microarray Analyses of the EI Stage

**Figure 2:**
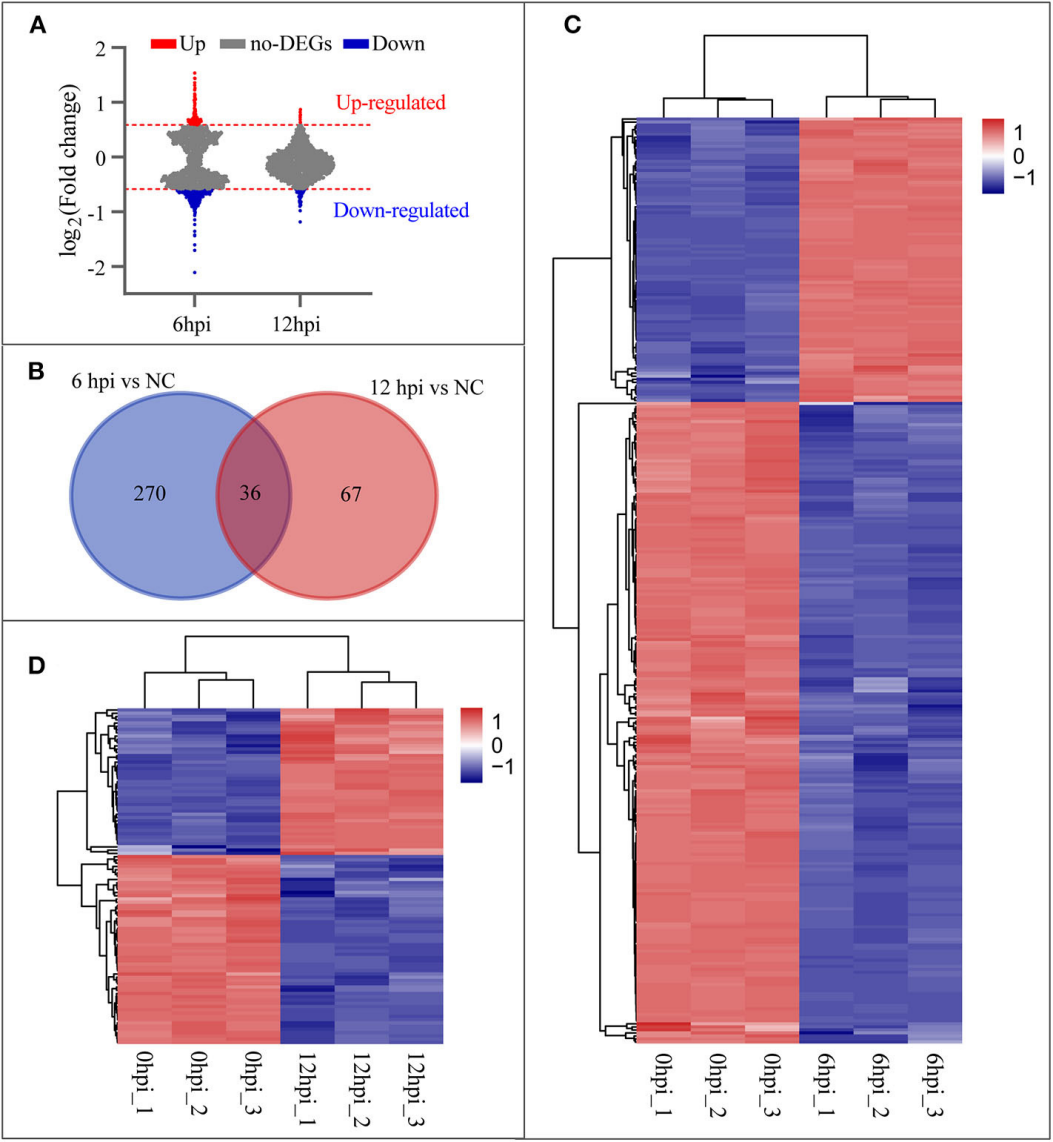
Differentially expressed genes (DEGs) at 6 and 12 hpi. **(A)** Changes of *B. pseudomallei* regulated cellular genes over time compared with NC (0 hpi) during the early stage of infection. Counts of genes up-regulated or down-regulated are shown in red or light blue dots, respectively, and a cut-off level of 1.5-fold change and a *P*-value < 0.05 were applied. **(B)** Venn diagrams depict the overlap of DEGs from each pairwise comparison between the time points, 6/0 hpi and 12/0 hpi. **(C,D)** Hierarchical clustering of DEGs at 6 and 12 hpi. Genes with high expression level are in red; genes with low expression level are in blue.

To investigate the host cellular factor involved in *B. pseudomallei* infection, we analyzed the mRNA by means of Affymetrix Human 2.0 Gene Chips at two time points (6 and 12 hpi) during the early stage of infection. Cells without infection (0 hpi) were used as the reference group for normalization. Applying a cut-off level of 1.5-fold change and a *P*-value < 0.05 on the mRNA level, we found that the cellular transcriptome profiles of duplicates were highly similar but diverged between time points, and that the number of DEGs at 6 hpi was greater than that at 12 hpi (Figure 2A). Figure 2B shows that the venn diagrams depict the overlap of DEGs from the time points of 6 and 12 hpi during the early stage of infection. A set of 306 genes at 6 hpi (Supplementary Table 3) and 103 genes at 12 hpi (Supplementary Table 2) compared with the uninfected groups (0 hpi) were observed. Thirty-six genes were involved in all two categories that indicated the 36 DEGs varied over time during the EI stage. The hierarchical clustering was performed for 306 genes (93 genes up-regulated and 213 genes down-regulated) at 6 hpi and 103 genes (45 genes up-regulated and 58 genes down-regulated) at 12 hpi, using the Pearson correlation as a similarity matrix. The heat-maps were generated to visualize the quantitative differences in the expression levels of DEGs at 6 and 12 hpi compared with 0 hpi. Clustered samples in the columns and the highly up-regulated (red) and down-regulated (blue) genes in the rows were shown in Figures 2C,D, respectively.

### Cellular Processes and Pathways Regulated by *B. pseudomallei* Infection

**Figure 3:**
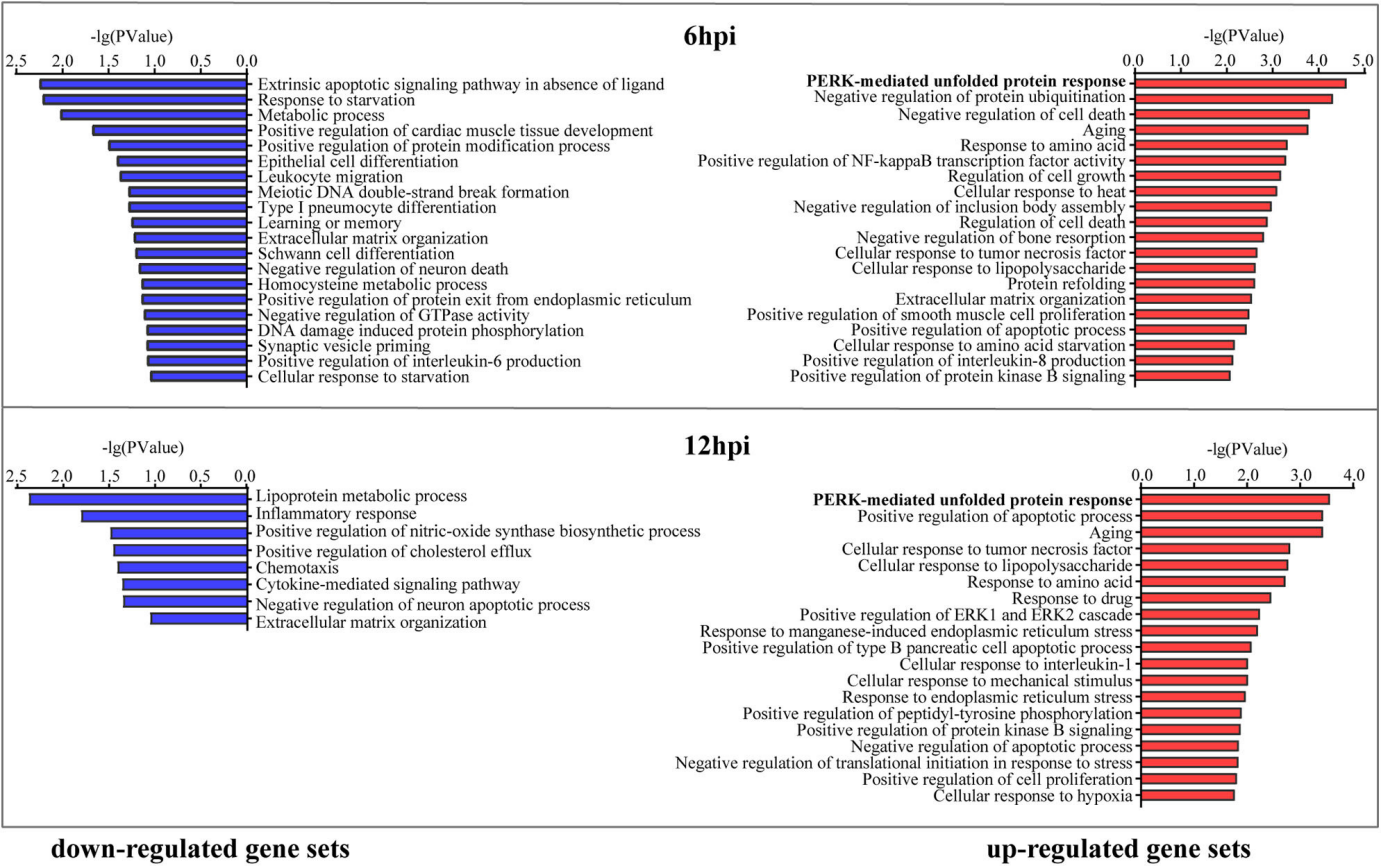
Cellular responses to *B. pseudomallei* infection analysis. Gene Ontology enrichment analysis was performed on up-regulated gene set and down-regulated gene set at 6 and 12 hpi, respectively. The significantly enriched Gene Ontology processes were ranked by their enrichment scores, and the top 20 processes were shown for each gene set. Blue and red panels represent down-regulated and up-regulated gene sets, respectively.

To explore cellular responses to *B. pseudomallei* infection and characterize DEG functions, we predicted enriched biological processes for up-regulated gene set and down-regulated gene set against the GO database, respectively, as a function of time following *B. pseudomallei* infection (Figure 3). No matter it was at 6 or 12 hpi, the most noteworthy enrichment biological process category was the PERK-mediated UPR with *P*-value = 2.48 × 10^−5^ at 6 hpi and *P*-value = 2.75 × 10^−4^ at 12 hpi in the up-regulated gene set, including three common DEGs of EIF2S1, HSPA5, also known as GRP78 or BiP, and ATF4. The expression levels of these three DEGs at later time points were comparable to or higher than those at earlier time points (Table 1), suggesting that PERK-mediated UPR was induced and became stronger over time during the EI stage.

**Table 1:** Differentially expressed genes of A549 infected with *Burkholderia pseudomallei* at 6 and 12 hpi in contrast to control cells (0 hpi).

**Gene**	**Functional category and protein name**	**mRNA accession**	**Fold change (vs. 0 hpi)**	
			**6 hpi**	**12 hpi**
**Unfolded protein response**				
EIF2S1	Eukaryotic translation initiation factor 2, subunit 1 alpha	NM_004094	1.72	1.71
HSPA5	Endoplasmic reticulum chaperone BiP	NM_005347	1.65	1.54
ATF4	Cyclic AMP-dependent transcription factor ATF-4	NM_001675	1.59	1.71
**Inflammatory response**				
SPINK5	Serine protease inhibitor Kazal-type 5	NM_001127698	0.36	0.52
MAP2K6	Mitogen-activated protein kinase kinase 6	NM_002758	0.36	0.65
IL20RB	Interleukin-20 receptor subunit beta	NM_144717	0.54	0.61
THBS1	Thrombospondin-1	NM_003246	2.15	1.96
TNFAIP3	Tumor necrosis factor alpha-induced protein 3	NM_006290	2.70	2.32
IL6	Interleukin-6	NM_000600	2.72	2.12
IL1A	Interleukin-1 alpha	NM_000575	0.58	0.54
ALOX5AP	Arachidonate 5-lipoxygenase-activating protein	NM_001629	0.48	0.54
GKN2	Gastrokine-2	NM_182536	2.15	2.20
F3	Tissue factor	NM_001993	2.28	2.11
NFκB2	Nuclear factor NF-kappa-B p100 subunit	NM_001077494	1.58	1.64
**Cell migration**				
ANXA9	Annexin A9	NM_003568	0.57	0.42
CLDN7	Claudin-7	NM_001307	0.64	0.47
ICAM1	Intercellular adhesion molecule 1	NM_000201	1.86	1.50
UBASH3B	Ubiquitin-associated and SH3 domain-containing protein B	NM_032873	2.40	2.15
SAMD9L	Sterile alpha motif domain-containing protein 9-like	NM_152703	0.56	0.63
CTGF	Connective tissue growth factor	NM_001901	1.69	1.72
**Apoptotic process**				
GADD45A	Growth arrest and DNA damage-inducible protein GADD45 alpha	NM_001924	2.03	1.69
FOSL1	Fos-related antigen	NM_005438	2.20	1.99
MT1X	Metallothionein-1X	NM_005952	2.20	1.88
BCL2L15	BCL2-like 15	NM_001010922	0.63	0.63
IGFBP3	Insulin-like growth factor-binding protein 3	NM_001013398	1.83	1.63
**Regulation of transport**				
RAB26	Ras-related protein Rab-26	NM_014353	0.55	0.57
ANKRD1	Ankyrin repeat domain-containing protein 1	NM_014391	2.51	2.18
STC2	Stanniocalcin-2	NM_003714	2.82	2.44
DENND2C	DENN domain-containing protein 2C	NM_198459	1.89	1.78
PLD1	Phospholipase D1	NM_002662	0.60	0.67
**G protein-coupled receptor signaling pathway**				
PPYR1	Neuropeptide Y receptor type 4	NM_005972	0.42	0.62
GPRIN3	G protein-regulated inducer of neurite outgrowth 3	NM_198281	0.43	0.58
**Cytoskeletal protein binding**				
EPB41L4A	Band 4.1-like protein 4A	NM_022140	0.34	0.56
**Zinc ion binding**				
MMP7	Matrilysin	NM_002423	2.16	1.94
CA11	Carbonic anhydrase-related protein 11	NM_001217	0.62	0.55
**Methyltransferase activity**				
METTL7A	Methyltransferase-like protein 7A	NM_014033	0.26	0.67

Certainly, many enrichment biological processes of DEGs in both up- and down-regulated gene sets pointed to inflammatory response, which included the processes of cellular response to tumor necrosis factor (TNF), cellular response to lipopolysaccharide, positive regulation of interleukin-8 and interleukin-6 production, and cellular response to interleukin-1. The DEGs of SPINK5, MAP2K6, IL20RB, IL1A, and ALOX5AP down-regulated over time (↓) and THBS1, TNFAIP3, IL6, GKN2, F3, and NFκB2 up-regulated over time (↑) were involved in this category. Other enrichment cellular processes that varied over time were cell migration including the DEGs of ANXA9↓, CLDN7↓, ICAM1↑, UBASH3B↑, SAMD9L↓, and CTGF↑ and apoptosis including the DEGs of GADD45A↑, FOSL1↑, MT1X↑, BCL2L15↓, and IGFBP3↑. The functional category and DEGs that varied over time were listed in Table 1.

**Figure 4:**
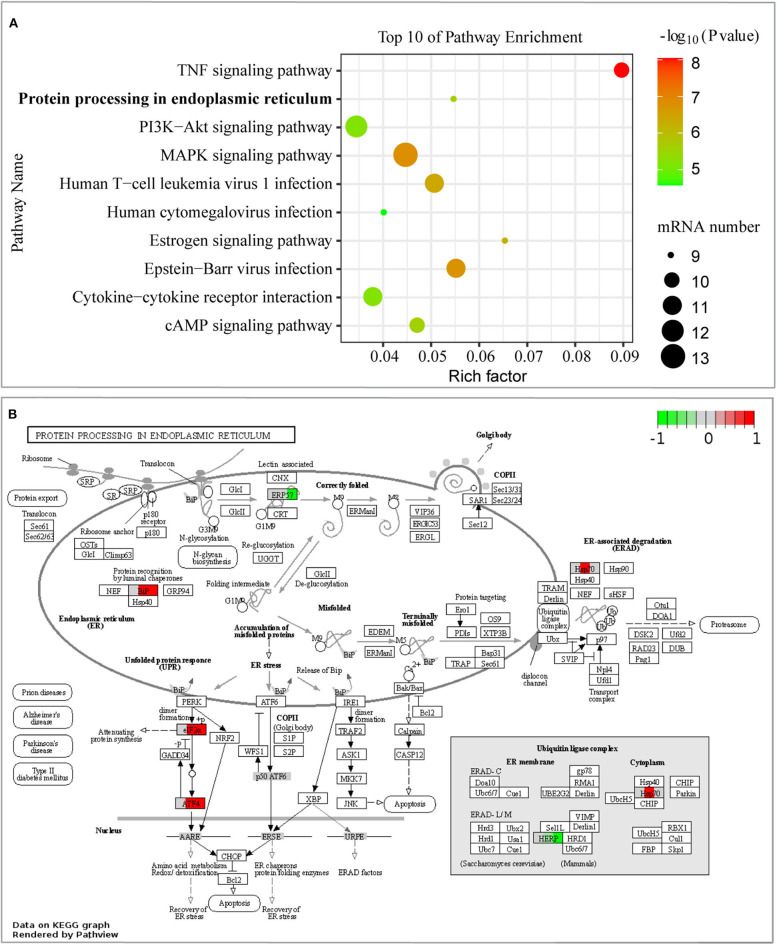
Pathway analysis of DEGs from GeneChip™ microarray assay. **(A)** The 10 most enriched KEGG pathways were displayed in scatter charts. Different colors indicate different *P*-value ranges. Important pathways were outlined in red bubbles. The different sizes of the dot indicate the number of target genes in the pathway. The pathway of protein processing in the endoplasmic reticulum was significantly enriched, *P* < 0.001. **(B)** Comparison of single gene expression changes in the pathway of protein processing in the endoplasmic reticulum. DEGs were colored in red (up-regulated) or green (down-regulated), and white or gray represents no significant difference in gene expression. Each colored box was divided into three parts, and the left, middle, and right parts represent the expression state of cells without infection, 6, and 12 hpi, respectively.

Moreover, we performed KEGG pathway classification for the DEGs in the early stage of infection, and it showed that the 10 top pathways were activated (Supplementary Table 5), including the signaling pathway of protein processing in the endoplasmic reticulum (ER) (Figure 4A). Differentially expressed proteins of BiP (also known as HSPA5/GRP78), eIF2α, ATF4, Hsp70 (including HSPA1A, HSPA1B, and HSPA2), HERPUD1, and EDEM2 were enriched in this signaling pathway, as shown in Figure 4B. What caught our attention was that the signaling pathway of protein processing in the ER was mainly induced by the branch of PERK-mediated UPR. The other top enrichment pathways were mainly involved in inflammatory response, including TNF signaling pathway and some viral-like pathogens infection signaling pathways, which were consistent with the results of GO enrichment analysis. In general, our results inferred that *B. pseudomallei* triggered the integration response of the host cell, but PERK-mediated UPR in the pathogenesis of *B. pseudomallei* should be paid more attention.

### Validation of Microarray Data and UPR

**Figure 5:**
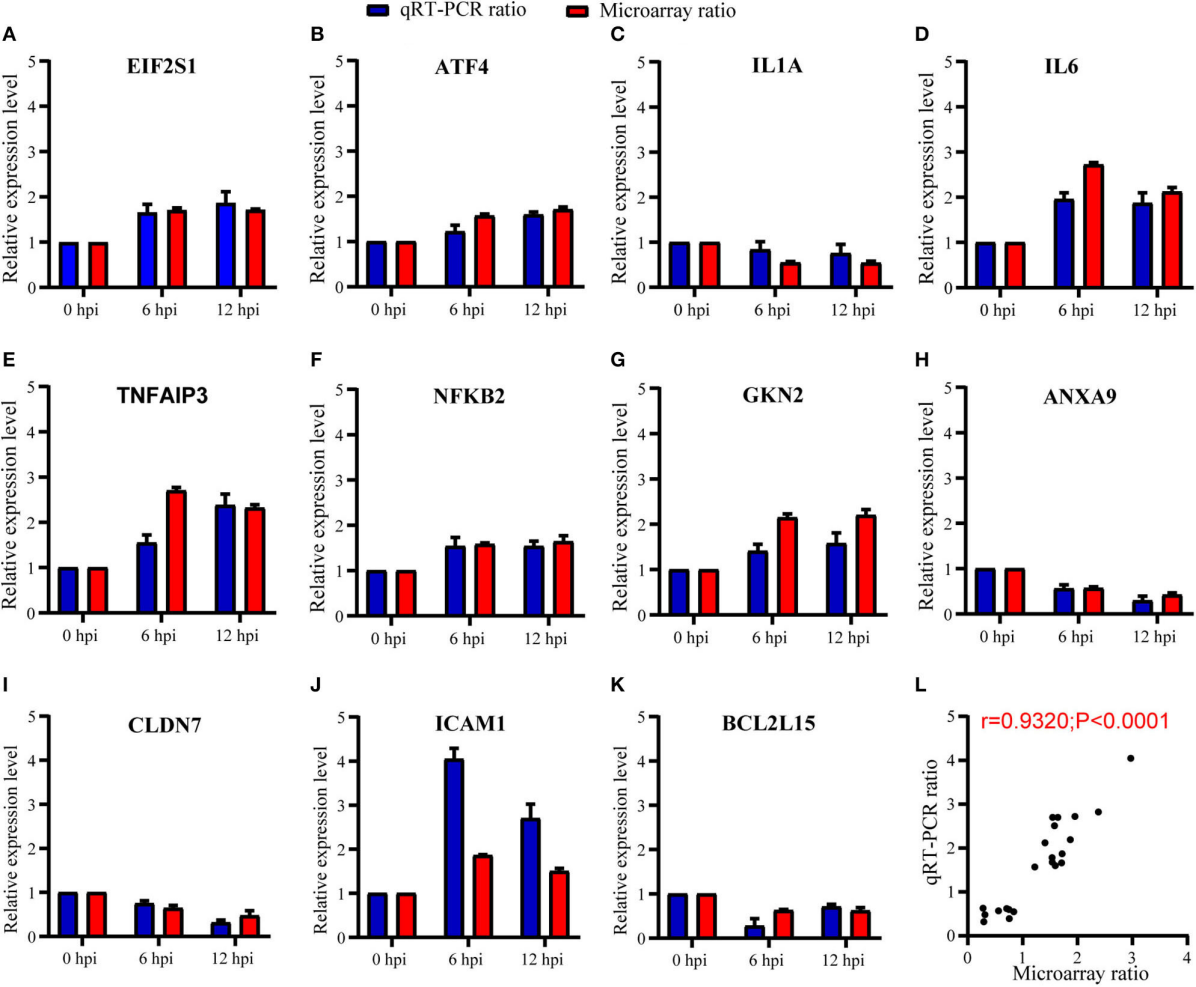
Expression level of host genes in A549 cells verified using RT-qPCR. **(A–K)** The relative expression level of each mRNA transcript was represented as the n-fold change relative to NC (0 hpi). EIF2α and ATF4 were verified in PERK-mediated unfolded protein response; IL1A, IL6, TNFAIP3, NFκB2, and GKN2 were verified in inflammatory responses; ANXA9, CLDN7, and ICAM1 were verified in cell migration; and BCL2L15 were verified in apoptosis. All data are presented as mean ± SD (*n* = 3 in each time point). **(L)** The correlation of expression level between GeneChip™ microarray and RT-qPCR was high and significant (Pearson's r = 0.93; *P* < 0.0001).

To validate the expression results observed in microarray, we further randomly selected 11 genes for verification by means of RT-qPCR. Selected genes were, respectively, involved in PERK-mediated UPR (eIF2α and ATF4), inflammatory responses (IL1A, IL6, TNFAIP3, NFκB2, and GKN2), cell migration (ANXA9, CLDN7, and ICAM1), and apoptosis (BCL2L15) in Table 1. As summarized in Figure 5, our RT-qPCR experiments indicated that the mRNA expression level of the 11 selected genes was consistent with the expression of microarray (Figures 5A–K), with the Pearson correlation r = 0.93 and *P*-value < 0.0001 (Figure 5L), which indicate that the microarray data were reliable.

**Figure 6:**
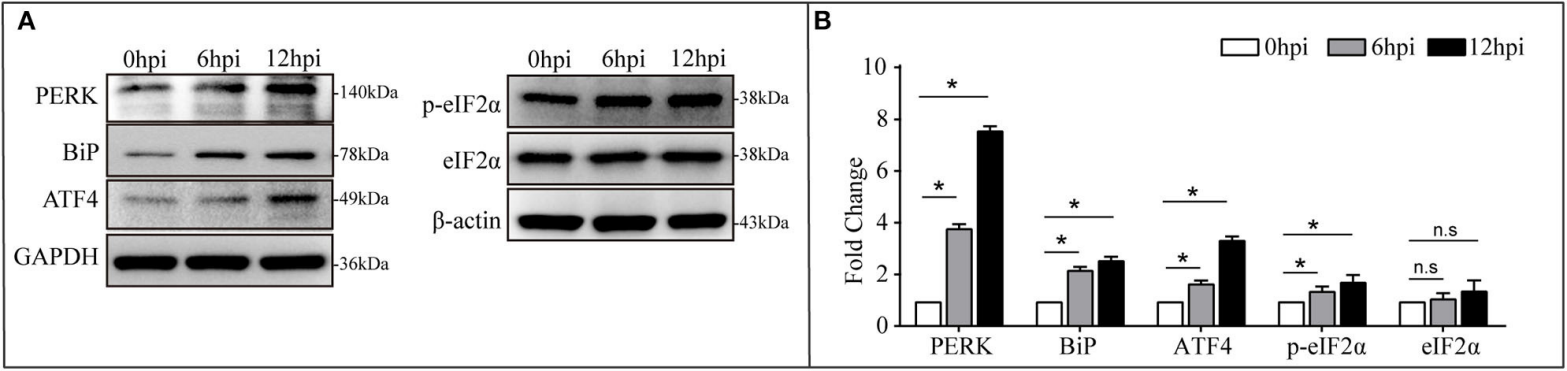
Expression of BiP (GRP78), PERK, eIF2α/p-eIF2α, and ATF4 was increased in *B. pseudomallei*-infected A549 cells. **(A)** Western blot analysis of DEGs involved in UPR challenged with *B. pseudomallei*. GAPDH and β-actin were used as loading controls, and all experiments were performed three times. **(B)** The relative expression level of DEGs in every group. Cells without infection (0 hpi) was the control group. A *t*-test was performed to compare the means, and the asterisks represent the *P*-value as follows * <0.05, n.s = non-significant, and *P*-value > 0.05 is considered not statistically significant.

The PERK-mediated UPR is one branch of UPR in the signaling pathway of protein processing in the ER (Bettigole and Glimcher, 2015). It will be activated to maintain protein-folding homeostasis in the ER. Concomitantly, mRNA translation was transiently attenuated through the phosphorylation of eIF2α leading to ATF4 activation (Hotamisligil, 2010). To investigate *B. pseudomallei*-activated PERK arm, BiP, PERK, eIF2α/p-eIF2α, and ATF4 were selected for verification *via* western blotting analysis, and the result was shown in Figure 6. With *B. pseudomallei* infection prolonged in the EI stage, the expression level of the ER chaperone proteins BiP, PERK, and ATF4 was verified to be significantly increased. The relative expression of BiP, PERK, and ATF4 was 2.20, 3.82, and 1.68 at 6 hpi, respectively, and then it went up to 2.58, 7.60, and 3.38 at 12 hpi, respectively. There was no significant change about the expression level of eIF2α, but the phosphorylation of eIF2α, the key to the activation of PERK-mediated UPR (Pavio et al., 2003), was verified to be significantly increased with the relative expression from 1.40 to 1.74. Our present data suggested that *B. pseudomallei* infection induced PERK-mediated UPR in human lung epithelial cells.

## Discussion

The intracellular lifestyle of *B. pseudomallei* in host cells has been reported to impact the severity of melioidosis, ranging from acute fatal sepsis to chronic infection with or without clinical symptoms (Pilatz et al., 2006; Lazar Adler et al., 2009; Allwood et al., 2011). However, the pathogenic mechanism of *B. pseudomallei* is still not fully understood. Elucidating the cellular response to *B. pseudomallei* infection will aid in unraveling the pathogenic mechanisms of the pathogen. We previously reported that *B. pseudomallei* could modulate autophagy (Li et al., 2015) and Rab32-mediated phagocytic vesicle transport to evade intracellular killing in host cells (Hu et al., 2019). Although previous studies have been carried out to determine the host transcriptional profiling of *B. pseudomallei* infection (Perumal Samy et al., 2015; Aschenbroich et al., 2019; Al-Maleki et al., 2020), limited by a narrow dynamic expression range, little is known about the dynamic changes of host response to *B. pseudomallei* infection. To further understand the cellular response dynamics caused by *B. pseudomallei*, we conducted a transcriptomic profiling of *B. pseudomallei*-infected A549 cells at 6 and 12 hpi during the EI stage. Our results generated a number of interesting observations. Upon *B. pseudomallei* infection, a set of 36 common genes that varied over time from 6 to 12 hpi were found. These DEGs are consistent with previous studies on the infection of *B. pseudomallei*, which involved inflammatory response (Wongprompitak et al., 2009; Perumal Samy et al., 2015), cell migration (Williams et al., 2014), apoptotic process (Hseu et al., 2014), and so on. The results indicated that these continuously regulated DEGs might be a sign of *B. pseudomallei* infection and played a key role in pathogenicity. Interestingly, the PERK-mediated UPR was enriched as the most noteworthy biological process category.

UPR is a cytoprotective response that prevents cytotoxicity effects caused by cellular accumulation of unfolded or misfolded proteins, which also invokes innate immune signaling in response to invading microorganisms (Celli and Tsolis, 2015). It is reported that various intracellular bacteria trigger the UPR to their advantage of replication during infection, such as *Mycobacterium tuberculosis* (Seimon et al., 2010), *Brucella melitensis* (Smith et al., 2013), and *Listeria monocytogenes* (Pillich et al., 2012). In our study, the expression of BiP, an ER chaperone protein, was increased as *B. pseudomallei* infection progresses, and *B. pseudomallei* infection increased PERK expression (Figure 6). This might be due to the accumulation of unfolded protein in the ER, resulting in cells needing more BiP to compensate for the dissociation of BiP from the transmembrane protein located in the ER, thereby helping the unfolding and assembly of protein structures (Bertolotti et al., 2000). Activated PERK could phosphorylate its target of the translation initiation factor eIF2α (Harding et al., 1999). Consistent with previous results, we confirmed that p-eIF2α increased with the time of *B. pseudomallei* infection. The p-eIF2α induced the activation of ATF4, which led to the transient weakening of gene translation involved in unfolded protein reaction (Choi and Song, 2019). The results showed that *B. pseudomallei* infection increased ATF4 expression (Figure 6). In conclusion, our present data suggested that *B. pseudomallei* induced PERK-mediated UPR in human lung epithelial cells. It was reported that UPR was exploited by energy and metabolically deficient pathogens to increase ATP and metabolites required for development, certain microbial agents, including viruses and bacteria, induce UPR (Celli and Tsolis, 2015), and it may be required for *Brucella* replication (Smith et al., 2013). Whether *B. pseudomallei* could hijack PERK-mediated UPR for intracellular survival and replication remains to be delved deeper. Moreover, the expression levels of up-regulated genes at later time points were comparable to or higher than those at earlier time points (Figure 6), suggesting that PERK-mediated UPR to *B. pseudomallei* infection became stronger over time. Although the UPR plays a major role in certain microbial infectivity, its role in *B. pseudomallei* pathogenesis is unknown. Other enrichment cellular processes, such as inflammatory responses, cell migration, and apoptosis, were enriched too. In general, our results inferred that *B. pseudomallei* triggered an integrated host cell response and the role of the UPR should be paid more attention to in the pathogenesis of *B. pseudomallei*.

Inflammatory response was also enriched according to microarray assay, as seen in Figures 3, 4. Microarray technology for transcriptome yields a large amount of data, and it is important to validate differential expression by independent methods, which conformed to the results of microarray assay. RT-qPCR analysis of DEGs included in inflammatory response, such as IL1A, IL6, TNFAIP3, NFκB2, and GKN2 (Figure 5), was therefore conducted. *B. pseudomallei* increased the mRNA expression of TNFAIP3 and IL-6 in human lung epithelial cell, which was consistent with the current research that proinflammatory mediators of TNF-α and IL-6 increased in the liver of mice following infection with *B. pseudomallei* and in serum samples of melioidosis patients (Ulett et al., 2000; Koosakulnirand et al., 2018). Activated UPR transcription factors can induce inflammatory response, which play an important role in the pathogenesis of inflammation (Bettigole and Glimcher, 2015). Previous studies showed that intracellular bacteria, such as *M. tuberculosis*, could induce p38 MAPK activation by TLR2 and TLR4, which in turn caused the accumulation of misfolded or unfolded TNF-α in the ER, and further induces the induction of macrophage ER stress (Oh et al., 2018; Choi and Song, 2019). Similarly, the MAPK signaling pathway was found in the process of *B. pseudomallei* infection in our manuscript (Figure 4A), and this might also be a possible way for *B. pseudomallei* to activate inflammatory response.

Additionally, our data yield that *B. pseudomallei* exposure could active the p-eIF2α/ATF4 pathway that was verified by western blotting in Figure 6. Accumulating evidences suggested that the p-eIF2α/ATF4 pathway mediated the induction of a gene expression program that referred to integrated stress response (ISR). ISR is a common adaptive pathway to restore cellular homeostasis, which could be triggered by integrating various types of stress signals, including ER stress, amino acid deprivation, virus infection, and oxidative stress (Harding et al., 1999; Zhang et al., 2002; Yerlikaya et al., 2008), while it is not clear whether this response benefits the host or the pathogen. However, considering the steady growth of intracellular *B. pseudomallei* at the early stage of infection in Figure 1C, it is likely that *B. pseudomallei* has evolved to modulate the ISR to its advantage during infection, but it still needs sufficient experimental evidence to reveal to what extent *B. pseudomallei* uses to exploit this intracellular niche to promote their replication. Additionally, the current evidence that bacteria may regulate UPR comes from the research on bacterial toxins. For instance, subtilase SubAB from Shiga-toxigenic *Escherichia coli* (STEC) can specifically degrade the ER chaperone BiP and further activated IRE1, ATF6, and PERK, the three known UPR signaling arms (Morinaga et al., 2008). *B. pseudomallei* has a strong virulence system (Wiersinga et al., 2006), whether UPR can be activated by it is not clear. Future work is likely to reveal to which novel mechanisms are employed by *B. pseudomallei* to manipulate UPR signaling.

## Conclusions

In conclusion, this study has elucidated the dynamic DEG profiles following *B. pseudomallei* infection of A549 cells during the EI stage. Interestingly, a set of 36 common genes varied over time were found, and the PERK-mediated UPR was enriched as the most noteworthy biological process category. We have verified the DEGs with RT-qPCR and further confirmed that *B. pseudomallei* induced the expression of Bip (GRP78), PERK, p-eIF2α, and ATF4, the key proteins involved in the UPR. Overall, our analysis suggests that *B. pseudomallei* infection induced the PERK-mediated UPR in protein processing in the ER. Therefore, the data reported here are valuable resources for practical use to further characterize the mechanisms that are potentially involved in the intracellular living of *B. pseudomallei*.

## Data Availability Statement

The datasets generated for this study can be found in online repositories. The names of the repository/repositories and accession number(s) can be found in the article/Supplementary Material.

## Author Contributions

CR, CM, and YX are fully responsible for the study, including the study design, data collection, data analysis and writing. MZ, SY, WY and ZH help with experiment implementation and data collection. JY and XC provide experimental technical support. LD provides research funding management and reimbursement support. XM provides guidance and supporting contribution. QL and YL bear the fund, design, writing and critically reviewing the intellectual content of the article. All authors contributed to the article and approved the submitted version.

## Conflict of Interest

The authors declare that the research was conducted in the absence of any commercial or financial relationships that could be construed as a potential conflict of interest.

## Supplementary Material

Supplementary Table 1 Analysis of transcriptome sequencing reads mapped to the K96243 genome.

Supplementary Table 2 Primer sequences for analysis of gene expression using RT-qPCR.

Supplementary Table 3 Summary of significant differentially expressed genes at 6 hpi.

Supplementary Table 4 Summary of significant differentially expressed genes at 12 hpi.

Supplementary Table 5 The enrichment of the top ten pathways.

Supplementary Table 6 Affymetrix microarray raw data.

## Abbreviations

MNGCs: multinucleated giant cells
EI: early infection stage
LI: late infection stage
RT-qPCR: real-time quantitative PCR
IL-1A: interleukin-1A
IL-6: interleukin-6
TNF-α: tumor necrosis factor-alpha
p-eIF2α: phosphorylation of the alpha subunit of eukaryotic initiation factor 2
ATF4: cyclic AMP-dependent transcription factor 4
BPC006: *B. pseudomallei* C006 strain
DEG: differentially expressed gene
GO: Gene Ontology
KEGG: Kyoto Encyclopedia of Genes and Genomes
WHO: World Health Organization.
