# Supplementary material for: Transcriptome Analysis Reveals Unfolded Protein Response Was Induced During the Early Stage of Burkholderia pseudomallei Infection in A549 Cells
Source: Front Genet. 2020 Dec 8;11:585203. doi: 10.3389/fgene.2020.585203 (PMC7753206; doi:10.3389/fgene.2020.585203)
Supplement: Supplementary Table 1 — Analysis of transcriptome sequencing reads mapped to the K96243 genome. [file Table_1.DOCX]

Table S1. Analysis of transcriptome sequencing reads mapped to the K96243 genome.

| **Terms** | **Burkholderia pseudomallei BPC006** | |
| --- | --- | --- |
|  | **ChrI**  **(NC_018527.1)** | **Chr II**  **(NC_018529.1)** |
| Total cleaned reads | 4,001,777 | 3,153,284 |
| Total mapped reads | 3,801,688 | 3,058,685 |
| Percentage of total reads mapped to K96243 (%) | 95% | 97% |
| Total CDS | 7,357 | |
| Reads mapped to CDS | 6,924 | |
| Percentage of CDS mapped (%) | 94% | |
