# Supplementary material for: Transcriptome Analysis Reveals Unfolded Protein Response Was Induced During the Early Stage of Burkholderia pseudomallei Infection in A549 Cells
Source: Front Genet. 2020 Dec 8;11:585203. doi: 10.3389/fgene.2020.585203 (PMC7753206; doi:10.3389/fgene.2020.585203)
Supplement: Supplementary Table 2 — Primer sequences for analysis of gene expression using RT-qPCR. [file Table_2.DOCX]

Table S2. Primer sequences for analysis of gene expression using RT-qPCR.

| Primer name | Sequence (5′ → 3′ ) | Product size (bp) | Source |
| --- | --- | --- | --- |
| EIF2S1(eIF2α)-F | GCTTCTTCCTTCTTGTCACCAGTT | 97 | This study |
| EIF2S1(eIF2α)-R | CCTGCTCCTACAGTTCCACATCA |  |  |
| ATF4-F | CCTTCACCTTCTTACAACCTCTTCC | 125 | This study |
| ATF4-R | GTAGTCTGGCTTCCTATCTCCTTCA |  |  |
| IL1A-F | TAGTGAGACCAACCTCCTCTTCT | 189 | This study |
| IL1A-R | AGACAAGTGAGACTCCAGACCTA |  |  |
| IL6-F | GTGAGGAACAAGCCAGAG | 181 | This study |
| IL6-R | CGCAGAATGAGATGAGTTG |  |  |
| TNFAIP3-F | GTTCCTCCTCTCCTACCAAG | 108 | This study |
| TNFAIP3-R | ACGATGAAGCAGTCCTGAT |  |  |
| NFκB2-F | AGGACGAGAACGGAGACA | 125 | This study |
| NFκB2-R | GTGGTTGGTGAGGTTGACA |  |  |
| GKN2-F | ACTTACTCCAGCACCTTCCTCTC | 148 | This study |
| GKN2-R | GTCTCCTGAACATTGCCACCATT |  |  |
| ANXA9-F | TTCAGCGTGGACAAGGAT | 171 | This study |
| ANXA9-R | TGCCTGTAGAGACTTCATCA |  |  |
| CLDN7-F | TCATCGTGGCAGGTCTTG | 186 | This study |
| CLDN7-R | CAGGACAGGAACAGGAGAG |  |  |
| ICAM1-F | TATGGCAACGACTCCTTCTC | 111 | This study |
| ICAM1-R | TGTCTCCTGGCTCTGGTT |  |  |
| BCL2L15-F | GCTATCATTGCTGGTCGCCTTC | 107 | This study |
| BCL2L15-R | GCTCCTGTCTGTCCCTTAATGGT |  |  |
| GAPDH-F | ACAACTTTGGTATCGTGGAAGG | 101 | Li Liang et al [1] , Qinggang Tian et al [2] |
| GAPDH-R | GCCATCACGCCACAGTTTC |  |  |
| ACTB-F | CGTGCGTGACATTAAGGA | 174 | This study |
| ACTB-R | AAGGAAGGCTGGAAGAGT |  |  |

[1] Liang L, Zhou W, Yang N, Yu J, Liu H. ET-1 Promotes Differentiation of Periodontal Ligament Stem Cells into Osteoblasts through ETR, MAPK, and Wnt/β-Catenin Signaling Pathways under Inflammatory Microenvironment. Mediators Inflamm. 2016;2016:8467849. Doi:10.1155/2016/8467849.

[2] Tian Q, Xiao Y, Wu Y, Liu Y, Song Z, Gao W, Zhang J, Yang J, Zhang Y, Guo T, Dai F, Sun Z. MicroRNA-33b suppresses the proliferation and metastasis of hepatocellular carcinoma cells through the inhibition of Sal-like protein 4 expression. Int J Mol Med. 2016 Nov;38(5):1587-1595. doi: 10.3892/ijmm.2016.2754. Epub 2016 Sep 27. PMID: 28026002.
