# Supplementary material for: Transcriptome Analysis Reveals Unfolded Protein Response Was Induced During the Early Stage of Burkholderia pseudomallei Infection in A549 Cells
Source: Front Genet. 2020 Dec 8;11:585203. doi: 10.3389/fgene.2020.585203 (PMC7753206; doi:10.3389/fgene.2020.585203)
Supplement: Supplementary Table 5 — The enrichment of the top ten pathways. [file Table_5.DOCX]

Table S5. The enrichment of the top 10 pathway of differential expression genes (DEGs).

| KEGG pathway | Pathway ID | Number of molecules | | P value  (Fisher test) |
| --- | --- | --- | --- | --- |
|  |  | Mapping | All |  |
| TNF signaling pathway | hsa04668 | 10 | 112 | 2.24E-06 |
| MAPK signaling pathway | hsa04010 | 13 | 295 | 6.25E-05 |
| Epstein-Barr virus infection | hsa05169 | 11 | 201 | 1.46E-04 |
| Human T-cell leukemia virus 1 infection | hsa05166 | 11 | 219 | 1.94E-04 |
| Estrogen signaling pathway | hsa04915 | 9 | 138 | 3.79E-05 |
| Protein processing in endoplasmic reticulum | hsa04141 | 9 | 165 | 4.91E-03 |
| cAMP signaling pathway | hsa04024 | 10 | 214 | 3.49E-04 |
| Cytokine-cytokine receptor interaction | hsa04060 | 11 | 294 | 3.07E-03 |
| PI3K-Akt signaling pathway | hsa04151 | 12 | 354 | 5.45E-03 |
| Human cytomegalovirus infection | hsa05163 | 9 | 225 | 7.70E-03 |
